# Recognition of Azole-Resistant Aspergillosis by Physicians Specializing in Infectious Diseases, United States

**DOI:** 10.3201/eid2401.170971

**Published:** 2018-01

**Authors:** Tiffany A. Walker, Shawn R. Lockhart, Susan E. Beekmann, Philip M. Polgreen, Scott Santibanez, Rajal K. Mody, Karlyn D. Beer, Tom M. Chiller, Brendan R. Jackson

**Affiliations:** Centers for Disease Control and Prevention, Atlanta, Georgia, USA (T.A. Walker, S.R. Lockhart, S. Santibanez, R.K. Mody, K.D. Beer, T.M. Chiller, B.R. Jackson);; University of Iowa Carver College of Medicine, Iowa City, Iowa, USA (S.E. Beekmann, P.M. Polgreen)

**Keywords:** Aspergillus fumigatus, aspergillosis, azole, pan-azole resistance, azole-naive, Cyp51A gene, voriconazole, antifungal, fungi

## Abstract

Infections caused by pan–azole-resistant *Aspergillus fumigatus* strains have emerged in Europe and recently in the United States. Physicians specializing in infectious diseases reported observing pan–azole-resistant infections and low rates of susceptibility testing, suggesting the need for wider-scale testing.

## Abstract 2

Of 709 physicians, 348 were familiar with azole-resistant *Aspergillus* *fumigatus*; of those treating case-patients, 21% lacked access to susceptibility testing.

Infections with strains of *Aspergillus fumigatus* that are resistant to all azole antifungal medications have become common in western Europe and have been documented in other regions since 1998 (*1*,*2*), but until recently, had not been reported in the United States (*3*). The Infectious Diseases Society of America recommends voriconazole, an azole antifungal medication, as first-line therapy for invasive *A. fumigatus* infections (*4*). Pan–azole-resistant *A. fumigatus* strains that have TR_34_/L98H and TR_46_/Y121F/T289A mutations of the *Cyp51A* gene have been associated with higher rates of treatment failure and death (*2*). These mutations may be linked to agricultural and environmental use of azole fungicides (*5*–*7*), because patients with these infections frequently lack prior clinical exposure to azoles (i.e., were azole-naive) (*2*). These persons were likely exposed to *A. fumigatus* that developed resistance after exposure to environmental fungicides. Limited genetic diversity between strains isolated from noncontiguous countries suggests a common origin with capacity for extensive geographic spread (*1*).

Recent limited data suggest a low rate of illness caused by azole-resistant *A. fumigatus* exists in the United States (*1*,*3*,*8*). Isolates with mutations conferring pan-azole resistance have recently been documented (*9*–*11*); however, little is known about the broader epidemiology, because there is no national surveillance of *Aspergillus* spp. In addition, little is known about the degree to which *A. fumigatus* resistance testing is available to US clinicians. These data could inform future testing and clinical practice.

## The Study

The Emerging Infections Network (EIN) surveyed US infectious disease physicians to better assess the availability of *A. fumigatus* susceptibility testing in clinical settings, the frequency with which clinicians request susceptibility testing, and the degree to which clinicians have observed azole resistance. The EIN is a provider-based emerging infections sentinel network supported by the Centers for Disease Control and Prevention and sponsored by the Infectious Disease Society of America (*12*). During May–June 2016, EIN distributed surveys to 1,584 members by email and fax; 709 (45%) responded.

Of the 709 respondents, nearly half (348, 49%) were familiar with the concept of azole-resistant *A. fumigatus*; 100 (14%) were aware of the possible link to agricultural or environmental antifungal products. During the previous year, 364 (51%) reported treating >1 patient who had been diagnosed with aspergillosis. Of those, 136 (38%) reported clinical failure of therapy for >1 patient, despite 290 (80%) physicians having used therapeutic drug monitoring to titrate azole therapy. Nine (2%) treating physicians reported observing azole resistance in an azole-naive patient.

Overall, 224 (62%) treating physicians who responded had access to susceptibility testing; 75 (21%) lacked access; and an additional 65 (18%) were unsure of availability. Of those with access, 182 (81%) reported that testing was physician-prompted; 162 (72%) reported that testing occurred off-site rather than in their hospital. For those reporting off-site testing, 8 (4%) physicians typically received results within 1 week and 42 (19%) reported receiving results ≥3 weeks after request, excluding the minority (n = 6, 4%), who were unsure. Of the 224 physicians who had access to susceptibility testing, 127 (57%) reported that >1 of their patients had an isolate tested, and 56 (25%) reported that >50% of patients had isolates tested. Forty-one (19%) reported a patient isolate with resistance to >1 azole, and 16 (7%) reported a patient with a pan–azole-resistant isolate.

Sixteen (8%) physicians practicing in the southern and 14 (9%) practicing in the western US census regions reported seeing >8 patients who had aspergillosis during the previous year, compared with 5 (3%) in the Northeast and 9 (5%) in the Midwest (χ^2^ = 6.3, p = 0.18). Other findings were generally similar across regions, including proportions reporting clinical failure, azole resistance in azole-naive patients, susceptibility testing availability, routine versus physician-prompted testing, and location of testing.

Of 224 physicians with access to susceptibility testing, 8 (16%) of 51 physicians in the South reported that >50% of their patients’ isolates were tested, compared with 17 (27%) of 63 in the Midwest, 8 (21%) of 37 in the Northeast, and 22 (31%) of 70 from the West (χ^2^ = 4.3, p = 0.37). Of 51 physicians in the South, 13 (26%) reported observing isolates resistant to >1 azole, compared with 9 (14%) of 63 from the Midwest, 5 (14%) of 37 from the Northeast, and 13 (19%) of 70 from the West (χ^2^ = 10.2, p = 0.04). Pan–azole-resistant isolates were reported by 4 (8%) of 51 physicians in the South, 7 (11%) of 63 in the Midwest, 2 (5%) of 37 in the Northeast and 3 (4%) of 70 in the West (χ^2^ = 4.4, p = 0.36).

In summary, approximately 50% (348/709) of surveyed infectious disease physicians were familiar with azole-resistant *A. fumigatus* and 14% (100/709) were aware of a possible link to environmental fungicide use. Of physicians who had treated patients diagnosed with aspergillosis within the past year, 21% (75/364) lacked access to susceptibility testing and 57% (127/224) who had access tested an isolate in the previous year. A small proportion of 19% (41/224) reported observing any azole resistance and only 7% (16/224) reported pan-resistance. Of note, physicians in the southern states more commonly observed resistance to >1 azole, compared with physicians from other regions.

Because only a small fraction of patients with invasive aspergillosis have a positive culture (*13*), a survey of resistance in culture-positive aspergillosis is not necessarily representative of all cases; but this fact highlights the importance of monitoring available cultures to inform broader practice. Another gap in our understanding of azole-resistant *A. fumigatus* is that the Clinical and Laboratory Standards Institute has not established breakpoints for azole susceptibility for *A. fumigatus* because inadequate clinical data exist to support breakpoints. The institute uses epidemiologic cutoff values, reflecting the minimal inhibitory concentration of 95% of wild-type isolates (*13*). However, there is some evidence that infection with resistant isolates by currently used thresholds is associated with worse outcomes in patients treated with azole monotherapy (*13*). Patients with hematologic or oncologic diseases are more likely to be infected with azole-resistant aspergillosis, and those with resistance have been shown to have higher case-fatality rates (*2*). However, it remains unclear to what degree these failures are attributable to underlying immunosuppression in these patients or to resistance-mediated treatment failure.

## Conclusions

Our findings support that azole-resistant *A. fumigatus* infections, including those with pan-azole resistance, are occurring in the United States, and that broader susceptibility testing may be warranted to guide patient care. Systematic surveillance for aspergillosis, including collection of clinical data and isolates, could aid in detecting emergence of regional resistance patterns, assessing the role that resistance plays in treatment failure, and determining locally tailored treatment options. Awareness by physicians of azole-resistant aspergillosis and the possible link to environmental fungicide use are essential.
